# Niche-Dependent Regulation of Lkb1 in the Proliferation of Lung Epithelial Progenitor Cells

**DOI:** 10.3390/ijms232315065

**Published:** 2022-12-01

**Authors:** Qingwen Ma, Xue Li, Sisi Wang, Qi Wang, Yu Li, Kuan Li, Jianhai Wang, Qiuyang Zhang, Junping Wu, Huaiyong Chen

**Affiliations:** 1 Department of Basic Medicine, Haihe Clinical School, Tianjin Medical University, Jinnan District, Tianjin 300350, China; 2 Department of Basic Medicine, Haihe Hospital, Tianjin University, Jinan District, Tianjin 300350, China; 3 Department of Tuberculosis, Haihe Hospital, Tianjin University, Jinnan District, Tianjin 300350, China; 4 Key Research Laboratory for Infectious Disease Prevention for State Administration of Traditional Chinese Medicine, Tianjin Institute of Respiratory Diseases, Jinnan District, Tianjin 300350, China; 5 Tianjin Key Laboratory of Lung Regenerative Medicine, Jinnan District, Tianjin 300350, China

**Keywords:** *Lkb1*, lung epithelial progenitor cells, proliferation, repair, autophagy, *Claudin-18*

## Abstract

Lung homeostasis and regeneration depend on lung epithelial progenitor cells. *Lkb1* (Liver Kinase B1) has known roles in the differentiation of airway epithelial cells during embryonic development. However, the effects of *Lkb1* in adult lung epithelial progenitor cell regeneration and its mechanisms of action have not been determined. In this study, we investigated the mechanism by which *Lkb1* regulates lung epithelial progenitor cell regeneration. Organoid culture showed that loss of *Lkb1* significantly reduced the proliferation of club cells and alveolar type 2 (AT2) cells in vitro. In the absence of *Lkb1*, there is a slower recovery rate of the damaged airway epithelium in naphthalene-induced airway epithelial injury and impaired expression of surfactant protein C during bleomycin-induced alveolar epithelial damage. Moreover, the expression of autophagy-related genes was reduced in club cells and increased in AT2 cells, but the expression of *Claudin-18* was obviously reduced in AT2 cells after *Lkb1* knockdown. On the whole, our findings indicated that *Lkb1* may promote the proliferation of lung epithelial progenitor cells via a niche-dependent pathway and is required for the repair of the damaged lung epithelium.

## 1. Introduction

The lung epithelium protects the lungs from environmental insults. The maintenance of lung tissue homeostasis and regeneration depend on healthy lung epithelial progenitor cells [1]. Two major types of epithelial progenitor cells are responsible for regenerating the airway and alveolar epithelial cells during lung injury. Club cells, as airway progenitor cells, can proliferate and differentiate into goblet and ciliated cells [2] at a steady state or after airway epithelial injury. Alveolar type 2 cells (AT2) synthesize and secrete numerous surfactant proteins that reduce alveolar surface tension and have antioxidant and antibacterial effects [3,4,5]. As alveolar progenitor cells, AT2 cells are capable of self-renewing and generating alveolar type 1 (AT1) cells at a steady state or after bleomycin (BLM)-induced lung injury [6,7,8]. Abnormal lung epithelial progenitor cell function leads to many respiratory diseases. Therefore, the regeneration of club and AT2 cells must be tightly regulated during lung homeostasis and damage.

Liver kinase B1 (*LKB1*), a tumor suppressor encoded by serine/threonine kinase 11 (*Stk11*), was first associated with Peutz-Jeghers syndrome and was involved in the control of embryonic development, tissue homeostasis, stem cell function, energy metabolism, and apoptosis [9,10,11,12,13,14,15,16,17,18]. The constitutive deficiency of *Lkb1* causes embryonic lethality and, in adult mice, contributes to weight loss and disrupted metabolism [16]. The regenerative capacity of hematopoietic stem cells in the bone marrow of irradiated mice decreases significantly after *Lkb1* deletion [7]. The loss of *Lkb1* in periosteal mesenchymal progenitor cells induces osteoblastogenesis by activating the mammalian target of rapamycin complex 1 (mTORC1) [19]. The loss of *Lkb1* in intestinal epithelial cells leads to impaired immune barriers and increased bacterial susceptibility [14]. *Lkb1* knockout in the renal epithelium activates chemokines and recruits inflammatory cells, leading to polycystic kidney disease [20]. *Lkb1* deletion promotes tumor cell proliferation by regulating mitogen-activated protein kinase (MAPK) signaling [21] and induces metabolic reprogramming [18] to promote tumorigenesis and lung cancer progression. Furthermore, *Lkb1* is required for the normal ciliated cell differentiation in both embryonic and adult lungs [10]. Our previous research has shown that *Lkb1* deficiency in the embryonic lung epithelium results in weight loss and tamoxifen-induced death within 5 weeks. *Lkb1* regulates airway goblet cell metaplasia by mediating interactions between airway progenitors and macrophages [22].

In conclusion, our results suggested that *Lkb1* may promote club cell proliferation via an autophagy-dependent pathway, while promoting AT2 cell proliferation in an autophagy-independent manner. Taken together, our results proposed a niche-dependent regulatory mechanism by which *Lkb1* may affect airway and alveolar progenitor cell proliferation during homeostasis and lung injury.

## 2. Results

### 2.1. Distribution and Expression Levels of Lkb1 in Human and Mouse Lung Tissues

**Figure 1:**
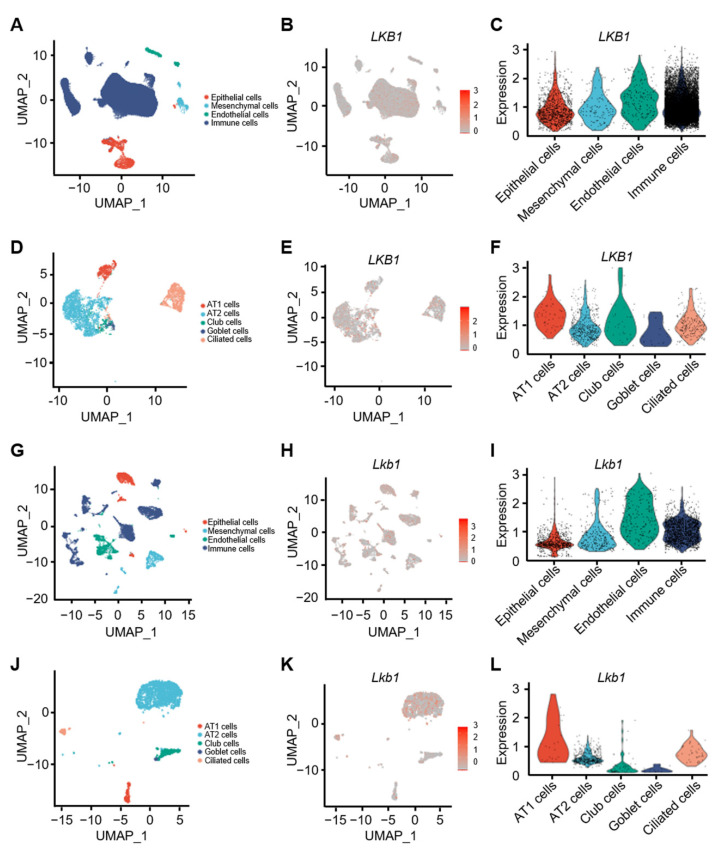
Distribution and expression levels of *Lkb1* in human and mouse lung tissues. (**A**) Uniform manifold approximation and projection (UMAP) plots of normal human lung tissues indicate four cell types, including epithelial cells, mesenchymal cells, endothelial cells, and immune cells. (**B**) Distribution of *LKB1* in human lung cell types. (**C**) Violin plots of *LKB1* expression levels in different lung cell types. (**D**) UMAP embedding of human lung epithelial cells. (**E**) Distribution of *LKB1* expression in human lung epithelial cells. (**F**) Violin plots of *LKB1* expression levels in different lung epithelial cells. (**G**) UMAP embedding of mouse lung tissue. (**H**) Distribution of *Lkb1* in mouse lung cell types. (**I**) Violin plots of *Lkb1* expression levels in lung cell types. (**J**) UMAP embedding of mouse lung epithelial cells. (**K**) Distribution and expression levels of *Lkb1* in mouse lung epithelial cells. (**L**) Violin plots of *Lkb1* expression levels in mouse lung cell types.

We have previously observed that the loss of *Lkb1* in the embryonic lung disrupts the lung epithelial structure and decreases the epithelial cell abundance in the adult lung [22]. Here, we investigated the mechanism by which *Lkb1* regulates the proliferation of lung epithelial progenitor cells in adult mice. Based on comprehensive analyses of published single-cell transcriptome sequencing data (GSE122960, GSE128033, GSE135893, and GSE136831) [23,24,25,26], we detected *LKB1* expression in various cell populations (epithelial, mesenchymal, endothelial, and immune cells) in normal human lung tissues (Figure 1A–C). We further observed the distribution and expression of *LKB1* in lung epithelial cells (AT1, AT2, club, goblet, and ciliated cells) (Figure 1D–F). Similarly, we analyzed the distribution and expression of *Lkb1* in different cell types in mouse lung tissues, including lung epithelial cells (Figure 1G–L). These results suggested that *Lkb1* is widely distributed in lung tissues and a certain expression level is maintained in lung epithelial cells at a steady state.

### 2.2. Lkb1 Is Required for Lung Epithelial Progenitor Cell Proliferation In Vitro

**Figure 2:**
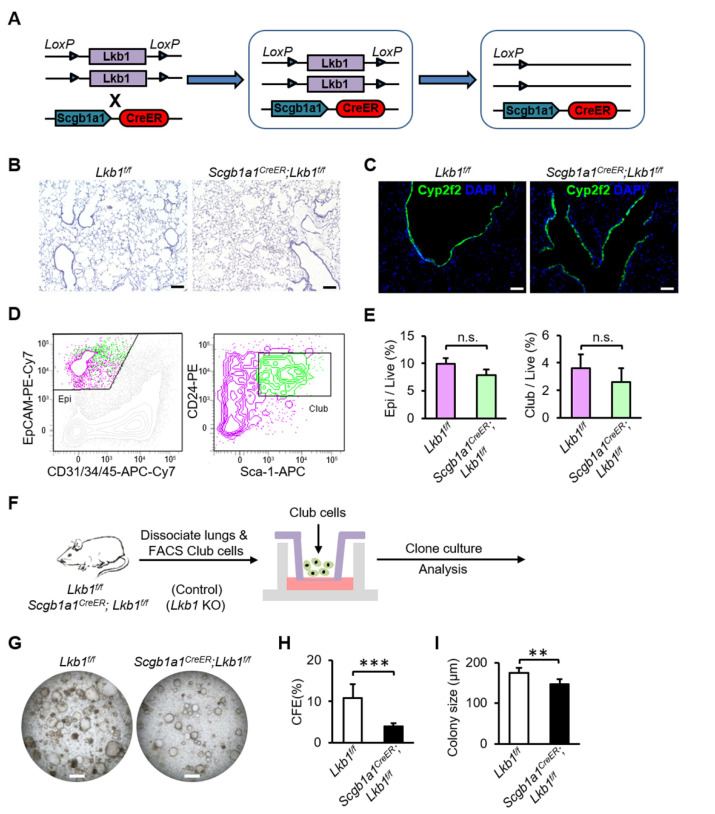
*Lkb1* is required for the proliferation of airway progenitor cells in vitro. (**A**) Schematic of *Scgb1a1^CreER^; Lkb1^f/f^* mice. (**B**) Hematoxylin and eosin staining of lung tissues from *Lkb1^f/f^* mice and *Scgb1a1^CreER^; Lkb1^f/f^* mice (Scale bars, 100 µm). (**C**) Immunostaining of lung tissues for the detection of *Cyp2f2* (Scale bars, 100 µm). (**D**,**E**) Flow cytometry showed that there were no obvious differences in the fraction of epithelial cells and the abundance of club cells in the total live cell population between *Scgb1a1^CreER^; Lkb1^f/f^* mice and *Lkb1^f/f^* mice. (**F**) Schematic diagram of methods for cell isolation and organoid culture of club cells. (**G**) Representative micrographs of organoid cultures of club cells isolated from *Lkb1^f/f^* mice and *Scgb1a1^CreER^; Lkb1^f/f^* mice (Scale bars, 500 µm). (**H**,**I**) CFEs (**H**) and colony size (**I**) of organoid cultures. Five independent experiments were conducted (n = 5). All data are presented as means ± SD, ** *p* ˂ 0.01; *** *p* ˂ 0.001.

As the main lung epithelial progenitor cells, club and AT2 cells are involved in the maintenance of lung epithelium function and repair after injury. Therefore, we constructed mice with a conditional deletion of *Lkb1* in airway club cells or alveolar AT2 cells to investigate the function of *Lkb1* in the regeneration of lung epithelial progenitor cells in adult mice. We created *Scgb1a1^CreER^; Lkb1^f/f^* mice by crossing *Lkb1^f/f^* mice with *Scgb1a1^CreER^* mice, in which *Lkb1* was conditionally deleted in airway club cells (Figure 2A). After the administration of tamoxifen to induce the loss of *Lkb1* in club cells, we did not detect obvious histological abnormalities in adult mice at a steady state (Figure 2B,C). To further evaluate the effect of *Lkb1* on the proliferation of club cells in vitro, we used an FACS-based method to separate mouse club cells (Figure 2D). Lin^−^EpCAM^+^Sca1^+^CD24^+^ club cells were sorted into organoid cultures. We observed no significant differences in the proportion of epithelial cells and abundance of club cells in the total live cell population after *Lkb1* deletion by flow cytometry (Figure 2E). Organoid culture indicated that the colony forming efficiency (CFE) (3.960 ± 0.779 (*Lkb1* KO) vs. 10.873 ± 3.261 (Control), *p* = 0.001) and colony size (146.200 ± 13.580 (*Lkb1* KO) vs. 175.591 ± 11.076 (Control), *p* = 0.003) of club cells sorted from *Scgb1a1^CreER^; Lkb1^f/f^* mice were obviously decreased (Figure 2F–I). These results suggested that *Lkb1* is essential for the proliferation of mouse club cells in vitro.

**Figure 3:**
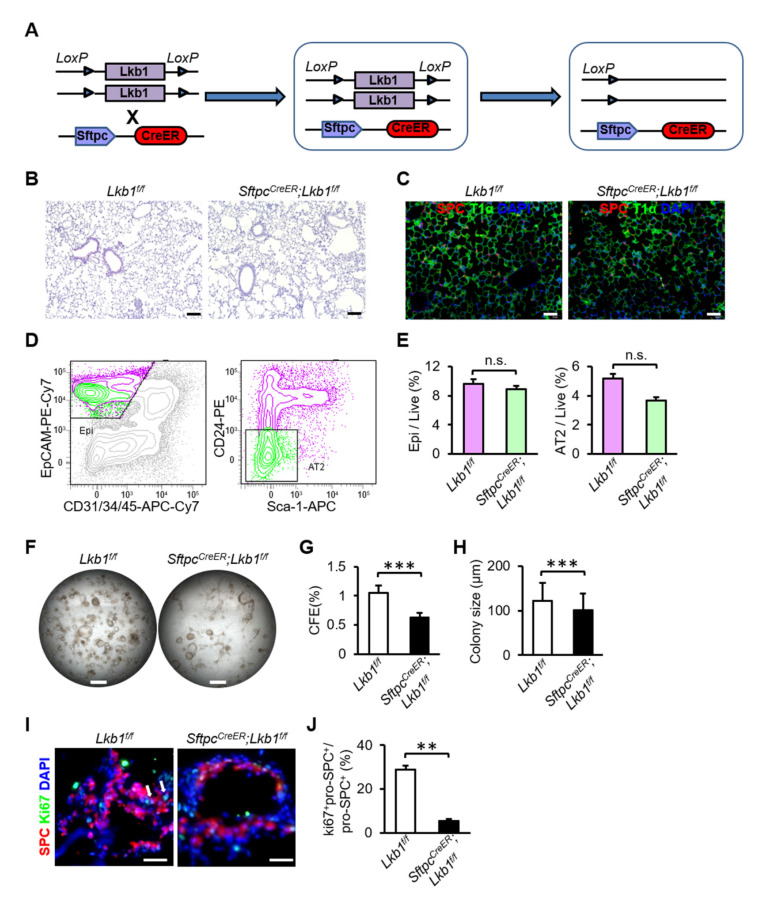
*Lkb1* is required for the proliferation of alveolar progenitor cells in vitro. (**A**) Schematic of *Sftpc^CreER^*; *Lkb1^f/f^* mice. (**B**) Hematoxylin and eosin staining of lung tissues from *Lkb1^f/f^* mice and *Sftpc^CreER^; Lkb1^f/f^* mice (Scale bars, 100 µm). (**C**) Immunostaining of lung tissues for SPC (AT2) and T1α (AT1) (Scale bars, 100 µm). (**D**,**E**) Flow cytometry showed no obvious differences in the fraction of epithelial cells and the abundance of AT2 cells in the total live cell population between *Sftpc^CreER^; Lkb1^f/f^* mice and *Lkb1^f/f^* mice. (**F**) Representative micrographs of organoid cultures of AT2 cells isolated from *Lkb1^f/f^* mice and *Sftpc^CreER^; Lkb1^f/f^* mice (Scale bars, 500 µm). (**G**,**H**) CFEs (**G**) and colony size (**H**) of organoid cultures. (**I**,**J**) Immunostaining of organoid culture and quantification of Ki67^+^SPC^+^ cells in total SPC^+^ cells in organoid cultures (Scale bars, 50 µm). Five independent experiments were conducted (n = 5). All data are presented as means ± SD, ** *p* ˂ 0.01; *** *p* ˂ 0.001.

To explore the effect of *Lkb1* on AT2 cell proliferation, we generated *Sftpc^CreER^; Lkb1^f/f^* mice with conditional loss of *Lkb1* in alveolar AT2 cells (Figure 3A). Tamoxifen was administered to mice aged 8–12 weeks by intraperitoneal injection to induce *Lkb1* deletion in alveolar AT2 cells. The lungs of *Sftpc^CreER^; Lkb1^f/f^* mice showed no significant histological abnormalities at a steady state (Figure 3B,C). To further analyze the effect of *Lkb1* on the proliferation of AT2 cells in vitro, we isolated AT2 cells from mouse lung tissues by FACS. The proportion of epithelial cells and the ratio of AT2 cells to total live cells did not differ between wild-type mice and mice with *Lkb1* deletion (Figure 3E). The CFE (0.637 ± 0.009 (*Lkb1* KO) vs. 1.050 ± 0.020 (Control), *p* = 0.001) and colony size (101.806 ± 36.761 (*Lkb1* KO) vs. 122.730 ± 39.257 (Control), *p* = 0.001) were reduced in the deletion of *Lkb1* (Figure 3D-H). Consistent with this, immunofluorescence staining of colonies showed that the fraction of Ki67^+^pro-SPC^+^ cells over total pro-SPC^+^ cells was lower (6.050 ± 0.522 (*Lkb1* KO) vs. 29.302 ± 7.109 (Control), *p* = 0.007) that in the absence of *Lkb1* (Figure 3I,J). Collectively, these results suggested that *Lkb1* is indispensable for mouse AT2 cell proliferation in vitro. Considering that the deletion of *Lkb1* may lead to the death of AT2 cells, we observed AT2 cell viability during organoid culture, and the results indicated that AT2 cells still survive (GFP^+^ cells), but the GFP^+^ cells could not proliferate normally to form clones after *Lkb1* deletion (Supplementary Figure S1A,B). On the other hand, bronchoalveolar lavage fluid (BALF) was added to the organoid cultures to evaluate the effect of growth factors in microenvironment on alveolar epithelial progenitor cell proliferation in vitro. Our results showed that reduced proliferation ability of AT2 cells caused by *Lkb1* deletion could not be rescued by BALF supplementation in organoid culture (Supplementary Figure S2A,B).

### 2.3. Lkb1 Is Beneficial for the Recovery of Lung Epithelium after Injury

**Figure 4:**
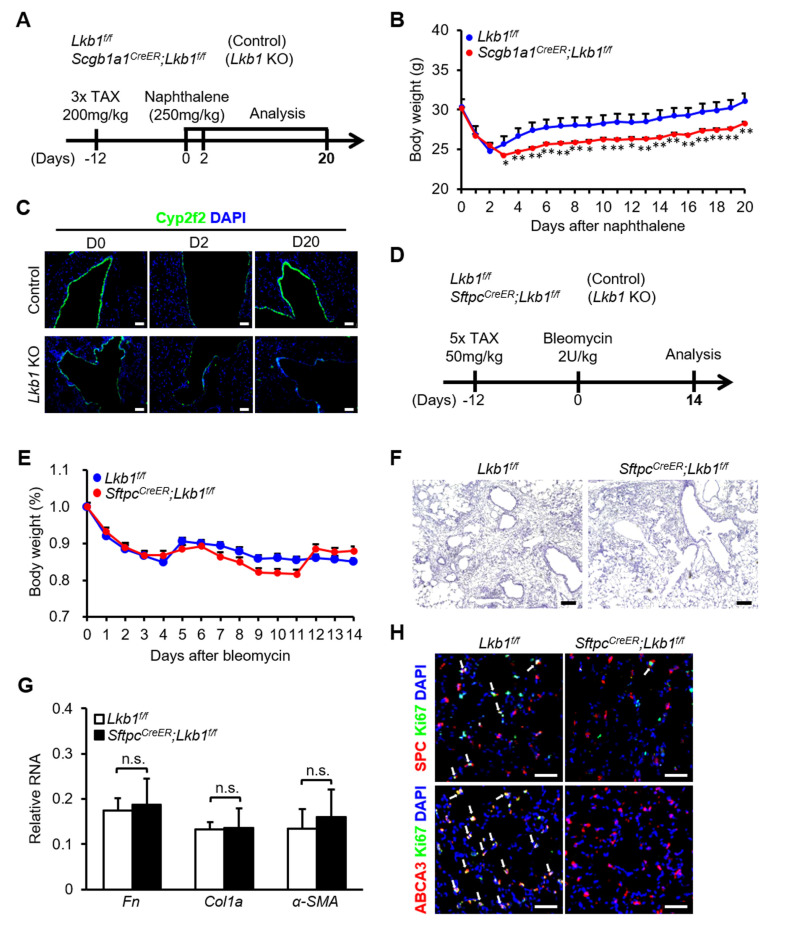
*Lkb1* is beneficial for the recovery of the lung epithelium after injury. (**A**) Naphthalene (250 mg/kg) was injected intraperitoneally to induce airway injury in *Lkb1^f/f^* mice and *Scgb1a1^CreER^*; *Lkb1^f/f^* mice and lung tissues were collected on days 0, 2, and 20 after injury (n = 10). (**B**) Body weight was analyzed after naphthalene treatment (n = 10). (**C**) Representative images showing the extent of club-cell injury or repair according to immunofluorescence: Cyp2f2 (green); DAPI (blue). (**D**) Bleomycin (2 U/kg) was administered to induce alveolar epithelium injury in *Lkb1^f/f^* mice and *Scgb1a1^CreER^*; *Lkb1^f/f^* mice and lung tissues were collected on day 14 (n = 10). (**E**) Body weight was obtained 14 days after BLM injury (n = 10). (**F**) Hematoxylin and eosin staining of lung tissues from *Lkb1^f/f^* mice and *Sftpc^CreER^*; *Lkb1^f/f^* mice at day 14 after BLM treatment. (**G**) qPCR analysis of *Fibronectin*, *Col1α*, and *α-SMA* expression levels in lung tissues (n = 7). (**H**) Immunofluorescence staining for *SPC*, *Ki67*, and *ABCA3* in lung tissues isolated from *Lkb1^f/f^* mice and *Scgb1a1^CreER^*; *Lkb1^f/f^* mice. Three independent experiments were conducted. All data are presented as means ± SD, * *p* ˂ 0.05, ** *p* ˂ 0.01. Scale bars, 100 µm.

Furthermore, we explored the regeneration of lung epithelial progenitor cells after *Lkb1* deficiency during lung injuries in vivo. In both *Lkb1^f/f^* mice and *Scgb1a1^CreER^; Lkb1^f/f^* mice, 250 mg/kg naphthalene was injected intraperitoneally to induce airway epithelium injury, and lung tissues were collected on days 0, 2, and 20 after naphthalene administration (Figure 4A). The decrease in club cells and loss of body weight in *Scgb1a1^CreER^; Lkb1^f/f^* mice caused by naphthalene were more remarkable than those in *Lkb1^f/f^* mice (Figure 4B). Immunofluorescence staining of lung tissues for the detection of *Cyp2f2* showed a more severe airway injury and slower the rate of recovery of damaged airways in the case of the deletion of *Lkb1* (Figure 4C). The above results suggested that *Lkb1* may protect the lungs from naphthalene-induced damage and promote the repair of the damaged mouse airway epithelium.

Similarly, 2 U/kg Bleomycin (BLM) was injected intratracheally to induce alveolar epithelium injury in *Lkb1^f/f^* mice and *Sftpc^CreER^*; *Lkb1^f/f^* mice, and lung tissues were collected on day 14 after BLM administration (Figure 4D). The body weight loss and degree of pulmonary fibrosis did not differ between the two groups (Figure 4E,F). There were no significant differences in the expression levels of pulmonary fibrosis markers, including *fibronectin (Fn)*, *Col1α*, and *α-SMA*, between mice with and without *Lkb1* (Figure 4G). Immunofluorescence staining showed normal expression levels of *ABCA3* in lung tissue between wild-type mice and mice with *Lkb1* deletion, while the proliferation of AT2 cells and the expression of *SPC* were impaired in the lung tissue of *Sftpc^CreER^; Lkb1^f/f^* mice (Figure 4H). These results showed that the secretion of *SPC* from AT2 cells was normal, but the expression of *SPC* was impaired after *Lkb1* deletion. All the data suggested that *Lkb1* may be beneficial for the recovery of the mouse lung epithelium after lung injury.

### 2.4. Possible Role of Autophagy and Claudin-18 in Lkb1-Mediated Regulation of the Proliferation of Mouse Lung Epithelial Progenitor Cells

**Figure 5:**
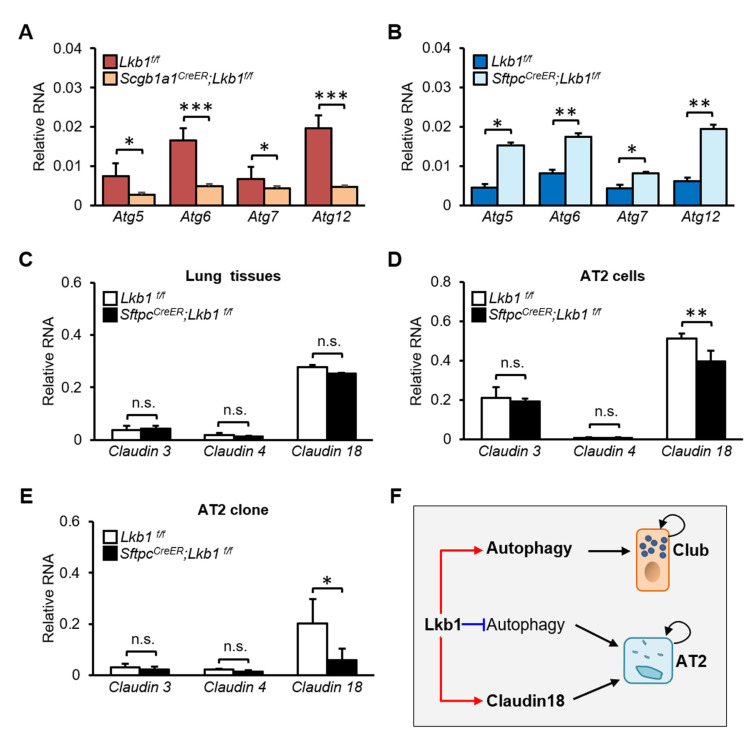
Possible role of autophagy and Claudin-18 in *Lkb1*-mediated regulation of the proliferation of mouse lung epithelial progenitor cells. (**A**) qPCR analysis of the expression of *Atg5*, *Atg6*, *Atg7*, and *Atg12* in club cells isolated from *Lkb1^f/f^* mice and *Scgb1a1^CreER^; Lkb1^f/f^* mice (n = 7). (**B**) qPCR analysis of the expression of *Atg5*, *Atg6*, *Atg7*, and *Atg12* in AT2 cells isolated from *Lkb1^f/f^* mice and *Sftpc^CreER^; Lkb1^f/f^* mice (n = 7). (**C**–**E**) qPCR analysis of *Claudin 3*, *Claudin 4* and *Claudin 18* expression in lung tissues (C), AT2 cells (D), and organoid cultures of AT2 cells E) isolated from *Lkb1^f/f^* mice and *Sftpc^CreER^; Lkb1^f/f^* mice (n = 6). (**F**) Schematic model describing the contribution of *Lkb1* to the proliferation of lung epithelial progenitor cells. Data are representative of three independent experiments. All data are presented as means ± SD. * *p* ˂ 0.05, ** *p* ˂ 0.01, *** *p* ˂ 0.001.

Autophagy is a conserved cellular process that can maintain the regenerative potential of the epithelium to response to stress [27]. We have previously reported that autophagy promotes the regeneration of mouse club cells and AT2 cells [1,28]. To further investigate whether *Lkb1* may regulate the proliferation of lung epithelial progenitor cells via autophagy, we isolated mouse club cells and AT2 cells using FACS, and RT-PCR was conducted to analyze the expression of *Atg*-related genes. We found that the levels of autophagy markers, including *Atg5* (0.003 ± 0.001 (*Lkb1* KO) vs. 0.007 ± 0.004 (Control), *p* = 0.013), *Atg6* (0.005 ± 0.001 (*Lkb1* KO) vs. 0.017 ± 0.002 (Control), *p* = 0.001), *Atg7* (0.004 ± 0.001 (*Lkb1* KO) vs. 0.007 ± 0.002 (Control), *p* = 0.042), and *Atg12* (0.005 ± 0.002 (*Lkb1* KO) vs. 0.020 ± 0.006 (Control), *p* = 0.001) were lower in club cells isolated from *Scgb1a1^CreER^; Lkb1^f/f^* mice than in those isolated from control mice (Figure 5A). These results suggested that *Lkb1* knockout may decrease the proliferation of club cells in vitro by affecting autophagy.

In a similar analysis of alveolar epithelial AT2 cells, the levels of autophagy markers, including *Atg5* (0.015 ± 0.006 (*Lkb1* KO) vs. 0.005 ± 0.002 (Control), *p* = 0.017), *Atg6* (0.017 ± 0.001 (*Lkb1* KO) vs. 0.008 ± 0.004 (Control), *p* = 0.003), *Atg7* (0.008 ± 0.002 (*Lkb1* KO) vs. 0.004 ± 0.002 (Control), *p* = 0.027), and *Atg12* (0.020 ± 0.004 (*Lkb1* KO) vs. 0.006 ± 0.002 (Control), *p* = 0.002), were higher in AT2 cells isolated from *Sftpc^CreER^; Lkb1^f/f^* mice (Figure 5B), contrary to the observations in club cells. Autophagy reprograms alveolar progenitor cell metabolism and is required for maintenance of the alveolar epithelium during lung injury [1]. We analyzed changes in genes associated with glutamine metabolism, glucose metabolism and lipid metabolism in AT2 cells (Supplementary Figure S3A–C, Table S1). The loss of *Lkb1* in AT2 cells had little effect on energy metabolism, and only decreased the expression of *got* (Supplementary Figure S3A). Additionally, we have reported that glutamine metabolism is required for alveolar regeneration during lung injury [29].

Claudins, as tight junction proteins, contribute to the proliferation and tumorigenesis of lung progenitor cells. *Claudin-18*, mostly expressed in alveolar epithelial cells [30,31], is rarely expressed in airways. We did not detect a change in the expression of *Claudin-18* in lung tissues, but observed decreased levels in both AT2 cells (0.396 ± 0.052 (*Lkb1* KO) vs. 0.512 ± 0.026 (Control), *p* = 0.004) and organoid cultures (0.060 ± 0.044 (*Lkb1* KO) vs. 0.201 ± 0.097 (Control), *p* = 0.023) after *Lkb1* deficiency (Figure 5C–E). Therefore, we hypothesized that *Claudin-18* may be involved in *Lkb1* knockout-induced reduction in the proliferation of mouse AT2 cells through affecting cell-cell adhesion/integrity in vitro. These findings suggested that *Lkb1* may promote the proliferation of mouse club cells and AT2 cells via different mechanism (Figure 5F).

## 3. Discussion

The pulmonary epithelium plays an essential role in gas exchange and host defense. As the dominant lung epithelial progenitor cells, club cells, and AT2 cells are responsible for maintaining homeostasis and repairing injuries [1,28]. In this study, we found that *Lkb1* is indispensable for the proliferation of normal lung epithelial progenitor cells in adult mice at a steady state. The loss of *Lkb1* significantly restricted the proliferation of club cells and AT2 cells, as validated by organoid culture in vitro. Additionally, in the absence of *Lkb1*, the recovery rate of the damaged airway epithelium was slower, and the secretion of *SPC* from AT2 was impaired. Furthermore, we observed altered expression levels of autophagy markers and *Claudin-18* in the mouse pulmonary epithelium with an *Lkb1* deficiency. Our results suggested that *Lkb1* may promote club cell proliferation via an autophagy pathway, but promote AT2 cell proliferation *via Claudin-18*. Our research will provide important insights into the mechanism of clinical treatment of *Lkb1*-related lung diseases.

*Lkb1*, a tumor suppressor, is strongly expressed in both human and mouse lung tissues. It is involved in the occurrence and progression of numerous diseases, especially in lung adenocarcinoma [32,33]. Tissue-specific *Lkb1* knockout studies have suggested that *Lkb1* contributes to stem cell regeneration and tissue homeostasis [19,34,35,36]. *Lkb1* directly activates MAPK to regulate cell survival, and *Lkb1*-deficient cells are highly sensitive to apoptosis caused by energy stress [17]. *Lkb1* deletion disrupts cell polarity and promotes collagen remodeling during tumor invasion [13]. Tang et al. demonstrated that the Lkb1/MARK3/ERK1/2 signaling cascade is a crucial regulator of ciliated cell fate and multiciliogenesis [10]. Our previous study revealed that *Lkb1* loss upregulates RELM-α in club cells, thereby regulating goblet cell differentiation metaplasia [22].

Autophagy is a conserved cellular process that maintains the regenerative potential of the epithelium in response to stress [27]. Autophagy affects the regenerative and therapeutic potential of mesenchymal stem cells [37]. Hematopoietic stem cells rely on autophagy to maintain normal metabolism and functions [38]. Aging muscle stem cells promote muscle regeneration via autophagy [39,40]. Hair follicle stem cells depend on autophagy to maintain their differentiation capacity [41]. Mice rely on autophagy to reduce excessive reactive oxygen species (ROS) and maintain the regeneration of intestinal stem cells [42]. Autophagy has been demonstrated to promote the regeneration of the airway epithelium and alveolar epithelium by metabolic reprogramming during pulmonary injury [1,28,29]. Similarly, our results suggested that *Lkb1* promotes club cell proliferation in an autophagy-dependent pathway, and the recovery rate of the damaged airway epithelium was slower in the absence of *Lkb1* in airway progenitor cells. The loss of *Lkb1* in AT2 cells decreased the expression of *got* associated with glutamine metabolism, which is required for alveolar regeneration during lung injury.

A growing body of research indicates that Claudins are involved in the regulation of cell proliferation and polarity. Claudins are tight junction proteins with a significant effect in lung progenitor cell proliferation and tumorigenesis [30]. *Claudin-18* is mainly expressed in lung alveolar epithelial cells [30,31], and is rarely expressed in airways. Other Claudins, such as *Claudin-3* and *Claudin-4*, are highly expressed in the airway epithelium. In mice with bleomycin-induced lung injury, the expression of genes encoding claudin proteins is reduced, especially *Claudin-18* [43], consistent with our results.

Our study had several limitations. First, organoid cultures showed significantly reduced proliferation of both club cells and AT2 cells in vitro; however, we did not detect an abnormal epithelial structure under homeostasis in vivo. We speculate that a compensatory mechanism in vivo attenuates the decreased cell proliferation caused by *Lkb1* deletion. Autophagy is the most likely mechanism; however, more research is needed to clearly explain the mechanism underlying the maintenance of homeostasis. Second, alveolar SPC secreted by AT2 cells reduces alveolar surface tension and increases lung compliance, thereby promoting the maintenance of a normal lung structure and function [44]. In this study, we observed a significant decrease in SPC expression after *Lkb1* deficiency during BLM-induced lung injury. Although it may prevent alveolar epithelium repair, we did not observe severe lung tissue damage in the absence of *Lkb1*. Third, we have no direct evidence to support the hypothesis that decreased autophagy directly influences airway epithelial cell proliferation in *Lkb1*-deficient mice. Claudin-18, as a tight junction protein and an important regulator of lung epithelial cell proliferation [45], showed decreased expression levels in AT2 cells and organoid cultures from *Lkb1*-deficient mice, suggesting that decreased Claudin-18 expression may affect the organoid formation through affecting cell proliferation and cell-cell junction. However, we lack direct evidence that reduced Claudin-18 expression affects alveolar epithelial cell proliferation through cell–cell adhesion in *Lkb1*-knockout mice. Therefore, more works should be focused on cell–cell adhesion/integrity and further experiments are needed to verify these results.

## 4. Materials and Methods

### 4.1. Mice

The experimental mice were retained in a specific pathogen-free (SPF) facility at Tianjin Haihe Hospital (SYXK (Jin) 2021–0002). The mice were exposed to a 12 h light/dark cycle and had free access to food and water. *Lkb1^f/f^* mice, originally from Dr. Ronald DePinho (Boston, MA, USA), were provided by Dr. Hongbin Ji (Shanghai Institute of Biochemistry and Cell Biology, Chinese Academy of Sciences, China). *Scgb1a1^CreER^* and *Sftpc^CreER^* mice were obtained from the Jackson Laboratory (Bar Harbor, ME, USA). To induce *Lkb1* knockdown in the airway epithelial progenitor cells, *Scgb1a1^CreER^* mice were crossed with *Lkb1^f/f^* mice to create *Scgb1a1^CreER^*; *Lkb1^f/f^* mice. Similarly, *Sftpc^CreER^*; *Lkb1^f/f^* mice were created by crossing *Sftpc^CreER^* and *Lkb1^f/f^* mice. The heterozygous mice were mated for 3–5 generations to obtain homozygous mice for experimental study. All experimental mice at 8–12 weeks of age were randomly assigned to groups. Mice were anesthetized using 1% sodium pentobarbital (50 mg/kg). All procedures involving animals were reviewed and approved by the Tianjin Haihe Hospital Animal Care and Use Committee (2021HHKT-018).

### 4.2. Naphthalene-Induced Airway Epithelium Injury

200 mg/kg tamoxifen (Sigma-Aldrich, St. Louis, MS, USA) was intraperitoneally injected into *Lkb1^f/f^* and *Scgb1a1^CreER^*; *Lkb1^f/f^* mice every other day for three times. After tamoxifen injection was completed, the mice rested for seven days and then were intraperitoneally injected with naphthalene (Sigma-Aldrich, St. Louis, MS, USA) dissolved in corn oil (250 mg/kg) (Sigma-Aldrich, St. Louis, MS, USA) to induce mouse airway epithelium injury. After naphthalene treatment, mouse lung tissues were collected on days 0, 2, and 20 for histological analyses.

### 4.3. Bleomycin-Induced Alveolar Epithelium Injury

*Lkb1^f/f^* and *Sftpc^CreER^; Lkb1^f/f^* mice received tamoxifen (50 mg/kg, i.p.) every day for five consecutive days to induce *Lkb1* knockout in mouse AT2 cells. Mice were anesthetized using 1% sodium pentobarbital and received an intratracheal injection of bleomycin (BLM) (Nippon Kayaku, Tokyo, Japan) at a dose of 2U/kg. Control animals only received phosphate-buffered saline (PBS) (Corning, Jiangsu, China). Lung tissues were separated for histological analysis on day 14 after BLM administration.

### 4.4. Mouse Lung Dissociation and Flow Cytometry

Mouse lung single-cell suspension was obtained as previously described [46]. Briefly, fresh isolated lung tissues were digested with elastase (Worthington Biochemical Corporation, Lakewood, NJ) and DNase I (Sigma-Aldrich) at 37 °C. Lung cells were resuspended in Hank’s balanced salt solution (HBSS) (Solarbio Beijing, China) with 2% FBS (Gibco, Thermo Fisher Scientific), 10 mM HEPES (Sigma-Aldrich), 0.1 mM EDTA (Invitrogen, USA), 100 IU/mL penicillin, and 100 g/mL streptomycin (Gibco, Thermo Fisher Scientific) and incubated with the following primary antibodies: CD31– biotin, CD34–biotin, CD45–biotin, CD24–PE, EpCAM–PE–Cy7, and Sca-1–APC. Cells were then incubated with streptavidin. 7-Amino-actinomycin D (7-AAD) was used to remove dead cells. All antibodies were purchased from eBioscience (San Diego, CA, USA). CD31^-^CD34^-^CD45^-^EpCAM^+^CD24^+^Sca-1^+^ club cells and CD31^-^CD34^-^CD45^-^EpCAM^+^CD24^-^Sca-1^-^ AT2 cells were sorted for organoid culture or RNA analyses.

### 4.5. Organoid Culture

Sorted club cells (5 × 10^3^ cells/well) or AT2 cells (2 × 10^4^ cells/well) were mixed with MLg fibroblasts in Matrigel (BD Pharmingen, San Diego, Calif)/basic medium (1:1) that contained DMEM/F12 (Corning, China), 10% FBS, 1% insulin-transferrin-selenium (ITS) (Sigma-Aldrich), 100 IU/mL penicillin, 100 µg/mL streptomycin and SB431542 (Sigma-Aldrich). The cell mixtures were then added to Transwell filter inserts (Greiner Bio-One, Kremsmunster, Austria) in 24-well plates containing 410 µL medium. Organoid cultures were maintained in an incubator with 5% CO_2_ at 37 °C, and the medium was renewed every other day. Organoid cultures were observed through an IX73 inverted fluorescence microscope (Olympus, Tokyo, Japan). Clones with diameters greater than 50 μm were counted, and colony-forming efficiency (CFE) was measured by counting the number of colonies in each well as a proportion of the implanted cells 10 days after seeding. Organoid cultures were embedded in Tissue-Ted optimal cutting temperature (O.C.T.) compound (Sakura, Torrance, Calif) for immunofluorescence or lysed for RNA analyses.

### 4.6. Hematoxylin and Eosin (H&E) Staining

As we described previously [1], Five μm lung slices were deparaffinized, rehydrated, and stained with hematoxylin (Zsqb-bio, Beijing, China) and eosin (Jiangyuan, Wuxi, Jiangsu, China) solution following the experimental protocol. Lung sections were then dehydrated with 95% and 100% alcohol. The slides were mounted with neutral resin.

### 4.7. Immunofluorescence Staining

Lung sections were sealed with 5% BSA (BOSTER, Wuhan, Hubei, China) and incubated with anti-CYP2F2 (1:200; Santa Cruz Biotechnology, USA), anti-surfactant protein C (SPC) antibody (1:200; Millipore, USA), anti-Podoplanin Monoclonal antibody (1:200; eBioscience, Thermo Fisher, USA), anti-Ki67 (1:100; eBioscience), and anti-ABCA3 (1:50; eBioscience). Samples were then incubated with the secondary antibody (1:200; Invitrogen, Carlsbad, Calif). Then, lung sections were mounted with Fluoromount G containing 4-6′-diami-dino-2-phenylindole (DAPI) (1:1000; Roche, Basel, Switzerland)). The sections were observed under a IX73 inverted fluorescence microscope.

### 4.8. RNA Extraction and qPCR

Total RNA was extracted from lung tissues or sorted lung epithelial progenitor cells using TRIzol reagent (Invitrogen) following the manufacturer’s instructions. 0.2 μg of total RNA was used for reverse transcription. Quantitative real-time PCR was performed using SYBR Green Supermix (Vazyme, China) and a Light Cycler 96 Real-Time PCR system (Roche Diagnostics, Indianapolis, IN). The PCR conditions were: 95 °C for 2 min, followed by 40 cycles of 95 °C for 10 s, 60 °C for 20 s, and 72 °C for 20 s. Gene expression was measured relative to the level of the endogenous reference gene, mouse β-actin. The primer sequences used for qPCR are shown in Table 1.

### 4.9. Single-Cell RNA Sequencing Analysis

The single-cell RNA sequencing data of human lung samples were downloaded from GEO database and analyzed using Seurat package on R platform. The cells expressed fewer than 3 genes, and the genes expressed in less than 200 cells were considered as low-quality cells and genes and removed from data matrices. The expression of genes was normalized using LogNormalize method: gene expression values for each cell were divided by the total number of transcripts of that cell and multiplied by 1000, and the results were then natural-log transformed using log1p. The cell types were annotated using canonical marker genes after dimension reduction and clustering based on KNN and SNN algorithm [47].

### 4.10. Statistical Analysis

All data are displayed as mean ± standard deviation (SD). Student’s *t*-tests were used to evaluate differences between the experimental and control groups. *P* < 0.05 was considered statistically significant (* *p* < 0.05; ** *p* < 0.01; *** *p* < 0.001).

## 5. Conclusions

In conclusion, our findings indicated that *Lkb1* may promote the proliferation of lung epithelial progenitor cells via a niche-dependent pathway, and is required for the repair of the damaged lung epithelium.

## Figures and Tables

**Table 1:** Sequences of primers for quantitative PCR.

Gene	Forward Primer	Reverse Primer
*β* *-actin*	5′-GGCCAACCGTGAAAAGATGA-3′	5′-CAGCCTGGATGGCTACGTACA-3′
*Fibronectin*	5′-GTGTAGCACAACTCCAATTACGAA-3′	5′-GGAATTTCCGCCTCGAGTCT-3′
*Col1a*	5′-CCAAGAAGACATCCCTGAAGTCA-3′	5′-TGCACGTCATCGCACACA-3′
*α* *-SMA*	5′-GCTGGTGATGATGCTCCCA-3′	5′-GCCCATTCCAACCATTACTCC-3′
*Atg5*	5′-TGAAAGAGAAGCAGAACCATACT-3′	5′-GGGTGTGCCTTCATATTCAAAC-3′
*Atg6*	5′-CCATCCTGGCGAGTTTCAATA-3′	5′-CCATCCTGGCGAGTTTCAATA-3′
*Atg7*	5′-TCCTGAGAGCATCCCTCTAAT-3′	5′-GGCTCGACACAGATCATCATAG-3′
*Atg12*	5′-TGAAGGCTGTAGGAGACACT-3′	5′-AGGCCACCAGTTTAAGGAAC-3′
*Claudin3*	5′-AACTGCGTACAAGACGAGAC-3′	5′- ACCAGGACACCGGTACTAA-3′
*Claudin4*	5′- GTGGCAAGCATGCTGATTATG-3′	5′- GAAGCCACCATAGGGTTGTAG-3′
*Claudin18*	5′- GGTATCCTCGTGTCCATCTTC-3′	5′-GATCCCAGAAGTCAGAGTCATC-3′

## Data Availability

The data used to support the findings of this study are included within the article.

## Supplementary Materials

The following supporting information can be downloaded at: https://www.mdpi.com/article/10.3390/ijms232315065/s1.
