# Supplementary material for: Niche-Dependent Regulation of Lkb1 in the Proliferation of Lung Epithelial Progenitor Cells
Source: Int J Mol Sci. 2022 Dec 1;23(23):15065. doi: 10.3390/ijms232315065 (PMC9735896; doi:10.3390/ijms232315065)
Supplement: Supplementary file 1 [file ijms-23-15065-s001.zip › ijms-2037039-supplementary.pdf]

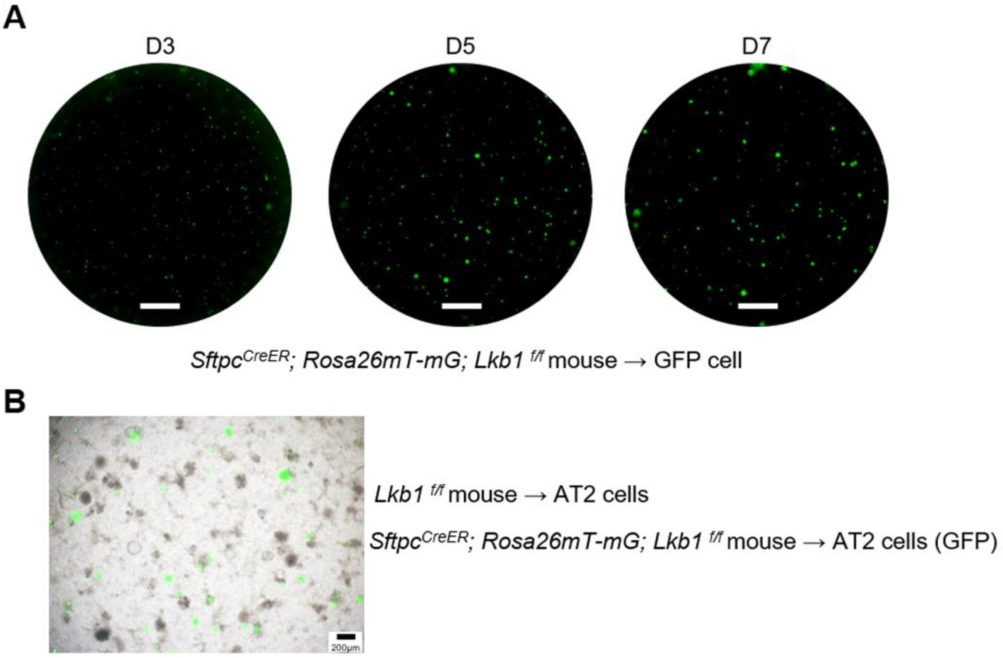

Figure S1. Viability of alveolar progenitor cells were unaffected in the absence of *Lkb1*. **(A)** Analysis of organoid cultures of AT2 cells (GFP) isolated from *Sftpc^CreER^; Rosa26^mT-mG^; Lkb1^f/f^* mice at different stages of growth. Scale bars, 500 μm. **(B)** A mixed organoid cultures of AT2 cells isolated from *Lkb1^f/f^* mice and *Sftpc^CreER^; Rosa26^mT-mG^; Lkb1^f/f^* mice. Scale bars, 200 μm. Three independent experiments were conducted (n=5).

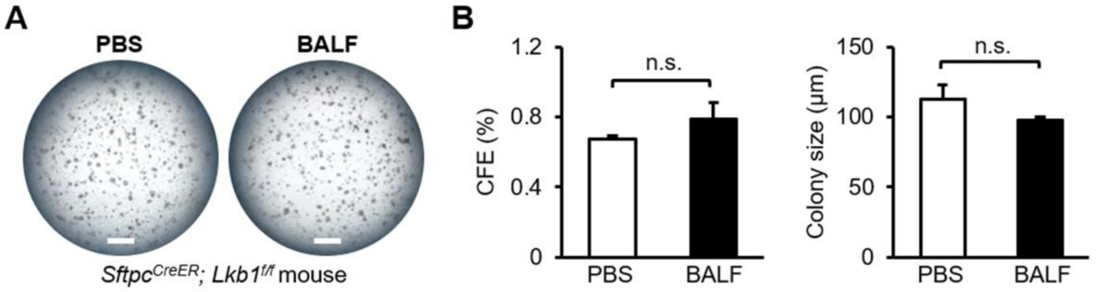

Figure S2. Bronchoalveolar lavage fluid could not rescue the inhibition of *Lkb1* deficiency on the proliferation of alveolar progenitor cells. **(A)** Organoid cultures of AT2 cells isolated from *Sftpc^CreER^; Lkb1^f/f^* mice with or without BALF (1:100). **(B)** CFEs and mean diameters of AT2 cells cultures. Three independent experiments were conducted (n=5). All data are presented as means ± SD. Scale bars, 500 μm.

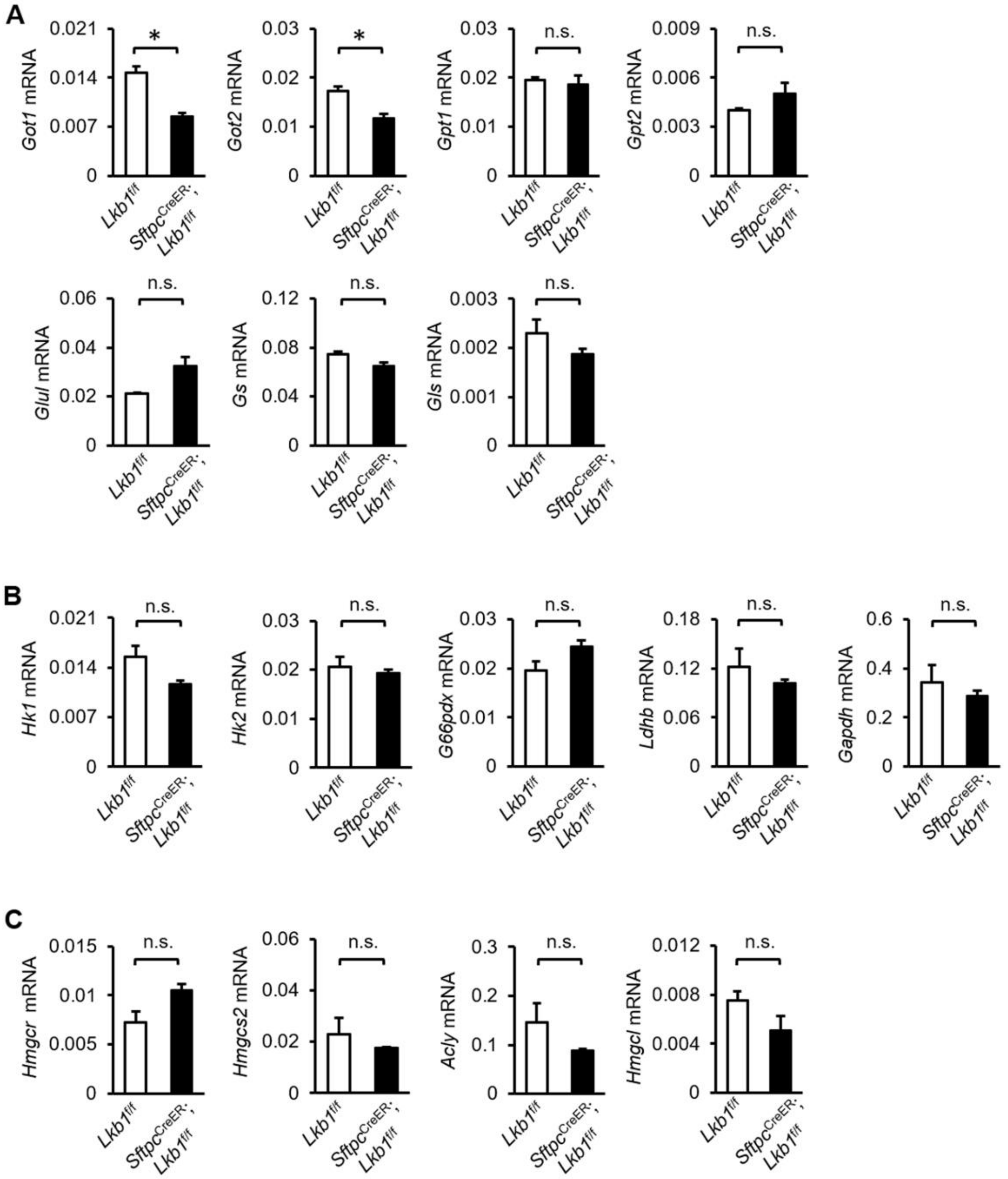

Figure S3. The change of energy metabolism of alveolar progenitor cells in the absence of *Lkb1*. **(A)** qPCR analysis of the expression of *Got1*, *Got2*, *Gpt1*, *Gpt2*, *Glul*, *Gs* and *Gls* genes related to glutamine metabolism. **(B)** qPCR analysis of the expression of *Hk1*, *Hk2*, *G6pdx*, *Ldhb* and *Gapdh* genes related to glucose metabolism. **(C)** qPCR analysis of the expression of *Hmgcr*, *Hmgcs2*, *Acly* and *Hmgcl* gene related to lipid metabolism. Three independent experiments were conducted (n=7). All data are presented as means ± SD. $^*p<0.05$. Abbreviations: Got1: glutamic-oxaloacetic transaminase 1; Got2: glutamatic-oxaloacetic transaminase 2; Gpt1: glutamic pyruvate transaminase 1; Gpt2: glutamic pyruvate transaminase 2; Glul: glutamate-ammonia ligase; Gs: greasy; Gls: glutaminase; Hk1: hexokinase 1; Hk2: hexokinase 2; G6pdx: glucose-6-phosphate dehydrogenase X-linked; Ldhb: Lactate dehydrogenase B; Gapdh: glyceraldehyde-3-phosphate dehydrogenase; Hmgcr: 3-hydroxy-3-methylglutaryl-Coenzyme A reductase; Hmgcs2: 3-hydroxy-3-methylglutaryl-Coenzyme A synthase 2; Acly: ATP citrate lyase; Hmgcl: 3-hydroxy-3-methylglutaryl-Coenzyme A lyase.

Table S1. Sequences of primers for quantitative PCR.

Gene	Forward primer	Reverse primer
β-actin	5′-GGCCAACCGTGAAAAGATGA-3′	5′-CAGCCTGGATGGCTACGTACA-3′
Got1	5′-CCCAAGCAGGTCGAGTATTT-3′	5′-TGGAGGTAGCGACGTAATCTA-3′
Got2	5′-TCGGTCTACATGACAAAGGATG-3′	5′-TACTTGGTGACCTGGTGAATG-3′
Gpt1	5′-ACTCAGTCTCTAAGGGCTACAT-3′	5′-CATCAGTTTCGCCATCTGTTTC-3′
Gpt2	5′-GCACCTACCCAAACCTACTAAA-3′	5′-GCCACATCTTCACGGATACA-3′
Glul	5′-CAAGTTCCCACTTGAACAAAGG-3′	5′-CGGTACCATCAACCCAGATATAC-3′
Gs	5′-GAGATCGCGACCTATCTGAAC-3′	5′-GTTACTGCCTTTGCCTTCTTTC-3′
Gls	5′-AGGGTGAAGTCGGTGATAAAC-3′	5′-GGGCTGTTCTGGAGTCATAAT-3′
Hk1	5′-CACTGATGGAGGTGAAGAAGAA-3′	5′-GGGATGCTCCGAACATAAGAA-3′
Hk2	5′-GCTGGAGGTTAAGAGAAGGATG-3′	5′-TGGAGTGGCACACACATAAG-3′
G6pdx	5′-CCACTCCAGAAGAAAGACCTAAG-3′	5′-TGGCTGTTGAGGTGCTTATAG-3′
Ldhb	5′-AGACAAGCTCAAAGGAGAGATG-3′	5′-CCGTCACCACCACAATCTTA-3′
Gapdh	5′-GGAGAAACCTGCCAAGTATGA-3′	5′-TCCTCAGTGTAGCCCAAGA-3′
Hmgcr	5′-CTGAAGGGTTTGCAGTGATAAAG-3′	5′-CCTGGACTGGAAACGGATATAG-3′
Hmgcs2	5′-CTGAAGGGTTTGCAGTGATAAAG-3′	5′-CCTGGACTGGAAACGGATATAG-3′
Acly	5′-CTCACACGGAAGCTCATCAA-3′	5′-TCCAGCATTCCACCAGTATTC-3′
Hmgcl	5′-AGATGGGTGTGAGTGTTGTG-3′	5′-ATGTAGACCAGGTCCTCAGTAG-3′
